# Frontal interhemispheric structural connectivity, attention, and executive function in children with perinatal stroke

**DOI:** 10.1002/brb3.2433

**Published:** 2021-11-25

**Authors:** Nicole Larsen, Brandon T. Craig, Alicia J. Hilderley, Shane Virani, Kara Murias, Brian L. Brooks, Adam Kirton, Helen L. Carlson

**Affiliations:** ^1^ Calgary Pediatric Stroke Program Alberta Children's Hospital Calgary Canada; ^2^ Hotchkiss Brain Institute University of Calgary Calgary Canada; ^3^ Alberta Children's Hospital Research Institute University of Calgary Calgary Canada; ^4^ Department of Pediatrics University of Calgary Calgary Canada; ^5^ Department of Clinical Neurosciences University of Calgary Calgary Canada; ^6^ Department of Psychology University of Calgary Calgary Canada; ^7^ Department of Radiology University of Calgary Calgary Canada

**Keywords:** ADHD, cerebral palsy, diffusion tensor imaging, executive function, perinatal stroke, tractography

## Abstract

Perinatal stroke affects ∼1 in 1000 births and concomitant cognitive impairments are common but poorly understood. Rates of Attention Deficit/Hyperactivity Disorder (ADHD) are increased 5–10× and executive dysfunction can be disabling. We used diffusion imaging to investigate whether stroke‐related differences in frontal white matter (WM) relate to cognitive impairments. Anterior forceps were isolated using tractography and sampled along the tract. Resulting metrics quantified frontal WM microstructure. Associations between WM metrics and parent ratings of ADHD symptoms (ADHD‐5 rating scale) and executive functioning (Behavior Rating Inventory of Executive Function (BRIEF)) were explored. Eighty‐three children were recruited (arterial ischemic stroke [AIS] *n* = 26; periventricular venous infarction [PVI] *n* = 26; controls *n* = 31). WM metrics were altered for stroke groups compared to controls. Along‐tract analyses showed differences in WM metrics in areas approximating the lesion as well as more remote differences at midline and in the nonlesioned hemisphere. WM metrics correlated with parental ratings of ADHD and executive function such that higher diffusivity values were associated with poorer function. These findings suggest that underlying microstructure of frontal white matter quantified via tractography may provide a relevant biomarker associated with cognition and behavior in children with perinatal stroke.

## Graphical abstract

Perinatal stroke affects ∼1 in 1000 births and concomitant cognitive impairments such as Attention Deficit/Hyperactivity Disorder (ADHD) and executive dysfunction can be disabling. We used diffusion tensor imaging (DTI) to investigate whether stroke‐related differences in frontal white matter (WM) relate to cognitive impairments. WM metrics correlated with parental ratings of ADHD and executive function suggesting that underlying microstructure of frontal white matter quantified via tractography may provide a relevant biomarker associated with cognition and behavior in children with perinatal stroke.

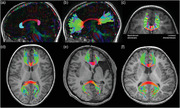

## 1 INTRODUCTION

Perinatal stroke is a cerebrovascular injury occurring in the fetus or newborn that affects ∼1 in 1000 births (Dunbar et al., 2020). Although predominately known for the subsequent sensorimotor deficits manifested as hemiparetic cerebral palsy, perinatal stroke may also lead to deficits in cognition and behavior (Bosenbark et al., 2018; Dunbar & Kirton, 2018; Fuentes et al., 2016; Kirton & deVeber, 2013; Murias et al., 2014; Westmacott et al., 2007). The two main subtypes of perinatal stroke are arterial ischemic stroke (AIS) and periventricular venous infarction (PVI) (Kirton et al., 2008). AIS results from an ischemic infarction, commonly involving the middle cerebral artery, that often damages cortical and subcortical areas (Kirton & deVeber, 2013). Neonatal arterial ischemic strokes are diagnosed soon after birth (following seizures), while arterial presumed perinatal ischemic strokes may be diagnosed later in infancy when early hand preference and hemiparesis become evident (Kirton & deVeber, 2013). These two AIS types only differ in timing of diagnosis (Dunbar & Kirton, 2019). By contrast, PVIs are venous infarctions resulting from a germinal matrix hemorrhage characterized by damage to the periventricular white matter. PVIs typically occur earlier than AIS, usually before 34‐week gestation (Kirton et al., 2008) and damage is restricted to the deep periventricular white matter. Both AIS and PVI result in deficits and will likely continue to occur given a lack of identified modifiable risk factors or prevention strategies (Dunbar & Kirton, 2018). Given that these focal injuries occur so early in life, studying children after perinatal stroke may give unique insight into neuroplastic compensatory mechanisms unfolding during development.

Perinatal stroke‐induced brain injury may lead to language, behavioral, and cognitive impairments (Bosenbark et al., 2018; Fuentes et al., 2016; Khan et al., 2021; Kirton & deVeber, 2013; Murias et al., 2014; Westmacott et al., 2007; Williams et al., 2018). Attention Deficit/Hyperactivity Disorder (ADHD) is a common neurodevelopmental disorder characterized by difficulty concentrating, overactivity, and impulsivity. Though ADHD may be perceived as a childhood disorder, it can persist well into adulthood often with academic, social, and occupational consequences (American Psychiatric Association, 2013). ADHD is one of the most commonly diagnosed disorders in children and those with perinatal stroke appear to be at a much higher risk (35–57%) than their peers (5–7%) (Bosenbark et al., 2018; Craig et al., 2018; Everts et al., 2008; Thomas et al., 2015; Westmacott et al., 2010; Westmacott et al., 2009; Williams et al., 2018). In addition to having a higher incidence of ADHD, children with perinatal stroke often have poorer cognitive and behavioral outcomes including poor inhibitory control, language delays, and diverse intellectual deficits (Fuentes et al., 2016; Hajek et al., 2013; Kirton & deVeber, 2013; Kirton et al., 2008; Lõo et al., 2018; Murias et al., 2014; Westmacott et al., 2010). The concept of central executive functioning encompasses such processes as sustained attention, inhibition, planning, and monitoring, particularly during novel problem solving and goal‐directed behavior. Deficits in executive function can be measured during formal neuropsychological assessments using standardized testing protocols, parental reports, and other validated assessment tools (Bosenbark et al., 2018; Everts et al., 2008; Ilves et al., 2016; Lõo et al., 2018; Westmacott et al., 2009).

How the nature of an early brain lesion interacts with developmental plasticity to produce variable outcomes in executive function and attention is not understood. Using MRI imaging techniques, differences in functionally connected neural networks of children with AIS compared to those with PVI and controls have been associated with cognitive function (Carlson et al., 2019; Ilves et al., 2016). Structural connectivity methods, including diffusion imaging (dMRI), allow for the isolation and characterization of specific white matter tracts in vivo. dMRI facilitates investigation of the diffusion of water across differing tissues within the brain and how larger molecules such as myelin, microtubules, and axons, restricts the movement of water (Basser, 1995; Beaulieu, 2002). Microstructural metrics such as fractional anisotropy (FA) and mean diffusivity (MD), among others, allow for quantification of the degree of diffusion in underlying tissue. Differences may reflect damage or differential development in white matter structures. These metrics as well as axial (AD) and radial diffusivity (RD) may provide additional information about developmental neuroplastic mechanisms following early brain injury. Our group and others have demonstrated the ability of tractography to better understand sensory and motor functions in children with perinatal stroke (Hodge et al., 2017; Kuczynski et al., 2017; Kuczynski et al., 2018; van der Aa et al., 2011). Furthermore, recent evidence has emphasized the essential role of widespread network alterations in the nonlesioned hemisphere in determining perinatal stroke outcomes (Craig et al., 2020), emphasizing the importance of interhemispheric connectivity.

Executive functions have largely been localized to areas of the frontal lobes using multimodal neuroimaging and subsequently, the importance of developing rich connectivity among frontal areas and other cortical, subcortical and limbic areas of the brain during maturation has been demonstrated (Fiske & Holmboe, 2019). Previous research using diffusion imaging metrics has shown an association between microstructural characteristics of frontal white matter, symptomology of ADHD, and executive function (Ashtari et al., 2005; Hong et al., 2014; Konrad & Eickhoff, 2010; Liston et al., 2011; Makris et al., 2009; Wu et al., 2020). Specifically, FA has been found to be lower in the frontal white matter of children with ADHD compared to peers (Konrad & Eickhoff, 2010; Liston et al., 2011; Tremblay et al., 2020; van Ewijk et al., 2012). Elevated MD in the anterior forceps of those with ADHD is a consistent finding across literature examining frontal tracts (Lawrence et al., 2013; van Ewijk et al., 2012). In adults, elevated MD was associated with longer reaction times during the Stroop task (a measure of cognitive interference), as well as decreased attention switching speed and flexibility (Mamiya et al., 2018). Findings related to other diffusivity metrics of the anterior forceps (radial [RD] and axial diffusivity [AD]) have been less consistent. AD tends to be higher in those with ADHD, but RD has shown mixed results (van Ewijk et al., 2012). In children with nonspecific unilateral cerebral palsy, differences in microstructure metrics in the anterior cingulate cortex have been associated with cognitive function (Scheck et al., 2015).

Given the high rates of ADHD and executive dysfunction in the perinatal stroke population, we investigated whether the character of frontal white matter in children with stroke would be altered compared to controls. We used dMRI tractography to examine the microstructure of the anterior forceps and possible relationships with parent ratings of ADHD and executive functioning. We hypothesized that the microstructure of frontal white matter would show disruptions (lower FA and higher MD, RD and AD) in children with AIS compared to children without injury (controls), and that children with PVI would be similar to controls given that cortical areas are typically preserved in this population. Further, we hypothesized that disruptions of frontal white matter (but not white matter in posterior lobes) would correlate with lower scores on executive function outcomes.

## 2 METHODS

### 2.1 Participants

Children with perinatal stroke were recruited from a population‐based research cohort, the Alberta Perinatal Stroke Project (APSP) (Cole et al., 2017). Inclusion criteria were (1) MRI‐confirmed unilateral perinatal stroke following term birth (> 36 weeks) with no evidence of additional bilateral or diffuse injury as confirmed by a pediatric neurologist and (2) MRI scan with anatomical and diffusion sequences taken between the ages of 6–19 years. We excluded participants with extreme head motion during the MRI scan (causing “venetian blind” artifacts that disrupted processing) or neurological conditions not attributable to the stroke. Control participants with no history of motor or neurological disorder were recruited through a community healthy controls program (HICCUP, www.hiccupkids.ca). Control participants were all right‐handed by self‐report and were similar in age (± 1 year) and sex to the stroke participants. Screening for ADHD and executive dysfunction was not performed in the control group; thus, this group likely reflected base rates found in a pediatric population with no neurological injury. This study was approved by the University of Calgary Research Ethics board. Parents gave written informed consent and eligible participants gave assent to take part in the study.

### 2.2 Image acquisition

Imaging was performed at the Alberta Children's Hospital using a 3 Tesla MRI scanner (GE MR750w, GE Healthcare, Waukesha, WI) with a 32‐channel head coil. All participants completed the standard research neuroplasticity protocol used at this site, which included a T1‐weighted (T_1_W) anatomical and diffusion (dMRI) scan. A fast‐spoiled gradient echo sequence was used to obtain high‐resolution T_1_W images axially (1 mm isotropic voxels, repetition time [TR] = 8.6 ms, echo time [TE] = 3.2 ms, flip angle = 11^o^, FOV = 256 mm, acquisition time = 5:10). An axial diffusion spin‐echo echo‐planar image scan was also acquired (32 diffusion directions; *b* = 0 s/mm^2^ [×3], 750 s/mm^2^; 2.5 mm isotropic voxels; 60 slices; TR = 11500 ms; TE = 69 ms; acquisition time = 6:45).

### 2.3 Image processing

Anatomical T_1_W scans underwent segmentation into cerebrospinal fluid (CSF), gray, and white matter using Statistical Parametric Mapping (SPM12; Wellcome Centre for Human Neuroimaging, UCL London). An estimate of total intracranial volume was calculated by summing volumes of CSF, gray, and white matter. Segmentations were also used to generate a gray matter–white matter interface mask, which was subsequently used to restrict the generation of reconstructed streamlines to only white matter, termed anatomically constrained tractography (Smith et al., 2012). Anatomical scans and masks were linearly transformed into diffusion space using FSL's “*FLIRT”* followed by nonlinear transformation using *“FNIRT”* (Andersson et al., 2007; Jenkinson et al., 2012).

The FSL FDT toolbox was used to correct eddy current and small head motion for the dMRI scan (Jenkinson et al., 2012). Color maps showing directionality of diffusion was calculated using MRtrix3's “*dwi2tensor”* command followed by *“tensor2metric”* (Tournier et al., 2019). Quality assurance was performed before and after color maps were generated, assessed by two researchers slice‐by‐slice axially (BTC and AJH). Reconstructed tracts were generated using MRtrix3's *“tckgen”* using the “*tensor_prob”* tracking algorithm (FA cutoff = 0.2; step size = 0.1 mm; angle threshold = 45°; 5000 streamlines; length range = 5–100 mm).

### 2.4 Regions of interest selection

**FIGURE 1:**
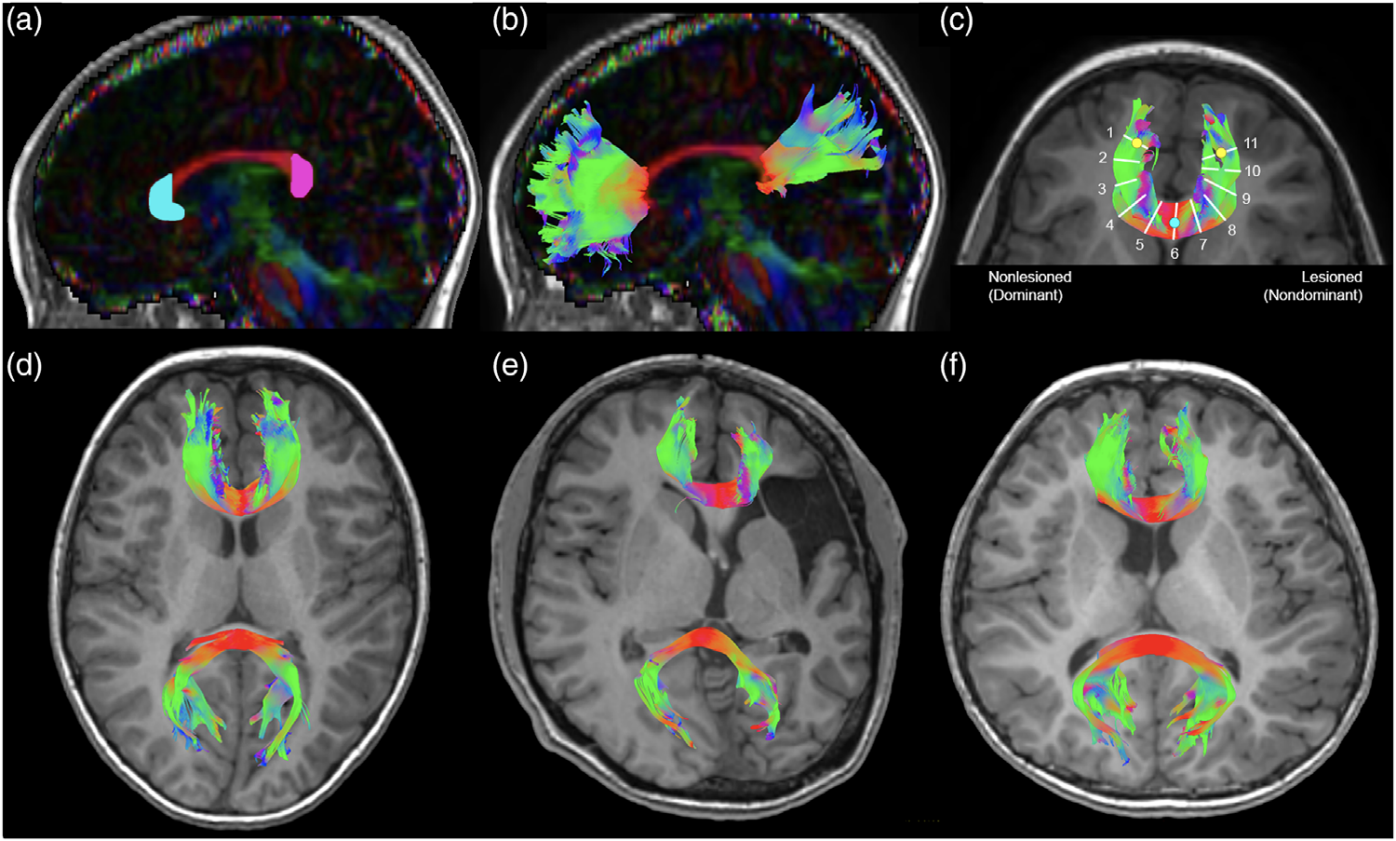
Region of interest placement and resulting representative anterior and posterior forceps tracts. (a) The color‐coded fractional anisotropy map was overlaid on the T_1_W anatomical image and a sagittal slice was selected at the midline where the corpus callosum is seen in full profile (red). Two regions of interest were drawn on the hooks of the genu (cyan) and splenium (magenta). (b) Resulting sagittal view of the reconstructed anterior and posterior forceps. (c) White matter metrics were measured from 11 tract samples along the anterior forceps along an arc defined using two anterior points (yellow circles) and one midline point (cyan circle). Representative tracts shown for a (d) controls, (e) AIS, and (f) PVI participant. Tract colors represent plane of projection. AIS, arterial ischemic stroke; PVI, periventricular venous infarction

Regions of interest (ROIs; Figure 1) were selected based on an anatomical model of the corpus callosum (Hofer & Frahm, 2006). A color map indicating directionality of water diffusion was overlaid on a T_1_W anatomical scan. In the axial view, the cursor was placed at the midline of the genu of the corpus collosum where the color map indicated primarily left‐right diffusion direction. In the sagittal view, the hook of the genu was then traced and filled to isolate the anterior forceps (Figure 1a).

The posterior forceps were also investigated for comparison and to assist in establishing functional specificity of subsets of the frontal white matter. A similar process was used to create the tracts for the posterior forceps, using an ROI placed over the splenium at the base of the posterior “bulb” of the corpus callosum at midline. Exclusion ROIs were drawn to demarcate spurious streamlines (typically the cingulum bundle) that were subsequently excluded during tract reconstruction.

For the anterior and posterior forceps separately, ROIs were used as seeds to select 5000 streamlines passing through them. This streamline threshold was chosen given previous findings of reliable white matter metrics using this degree of sampling (Reid et al., 2020). Resulting tracts were binarized using *“tckmap”* and then overlaid onto the tensor image where mean values of FA, MD, AD, and RD for the entire tract were extracted using *“tensor2metric”* and *“mrstats”* (Tournier et al., 2019).

### 2.5 Intrarater reliability

To assess the reliability of ROI placements and reconstruction of tracts, extraction of mean white matter metrics for the anterior forceps were repeated one month later by the same tractographer (NL) on a subset of 14 randomly selected participants (17% of the total sample).

### 2.6 Along‐tract white matter metrics

To investigate possible diaschisis (degeneration of brain structures spatially displaced from the primary lesion), white matter metrics were additionally measured at 11 points along the length of the reconstructed anterior forceps. The sampling points were manually determined by noting the coordinates of three reference points defining an arc covering the main body of the tract for each participant (in native space). Two anterior points of the forceps tract (yellow circles in Figure 1c) were identified by overlaying the forceps track reconstruction over the anatomical T1‐weighted image and locating the anterior‐most point in each frontal lobe projection before the tracts fanned out laterally/medially. The midpoint of this arc (cyan circle in Figure 1c) was identified by varying the opacity of the overlaid track file so that the underlying anatomical axial slice could also be seen while tract coordinates were noted where they crossed the midline. The *tckresample* function in MRtrix3 calculated locations for 11 equidistant points along the reference arc and using a line perpendicular to the tangent of the arc, white matter metrics were extracted from these 11 samples. Resulting white matter metric values were reassigned for children with left‐side stroke to match right‐side stroke such that nonlesioned/dominant (segments 1–5), midline (segment 6), and lesioned/nondominant (segments 7–11) were compared appropriately among groups.

### 2.7 Lesion volume

Lesion volumes (in cubic centimeters [cc]) were measured using the 3‐dimensional ROI selection tool in MRIcron (Rorden & Brett, 2000) based on T_1_W image intensity. For the AIS group, the center of the lesion was selected on an axial slice and the resulting dilated ROI was verified and adjusted manually (if necessary) on every axial slice. For the PVI group, bilateral ventricle volumes were measured as above and ventricle asymmetry (lesion size) was calculated as the absolute value of the difference between the two volumes as such: PVI lesion size = | lesioned hemisphere ventricle volume – nonlesioned ventricle volume |.

### 2.8 Cognitive function

Parents completed questionnaires examining executive function and symptoms of ADHD as part of a larger clinical neuropsychological assessment. The Behavior Rating Inventory of Executive Function (BRIEF) was used to assess executive function in children and adolescents (Gioia et al., 2000). It is a parental measure composed of 86 items comprising eight clinical scales. Three of the eight scales measure behavioral regulation (inhibit, shift, emotional control). The remaining five are related to metacognition (initiate, working memory, plan/organize, organization of materials, monitor) as well as an additional global executive composite score. The ADHD rating scale (ADHD‐5; DuPaul et al., 2016) assesses levels of ADHD symptomology over the previous 6 months. It is composed of 18 questions, half pertaining to inattention and half to hyperactivity. Higher scores on both questionnaires reflect a higher degree of dysfunction. Percentiles (for ADHD‐5) and T score values (for BRIEF) in relation to age‐matched normative peer groups are reported.

### 2.9 Statistical analysis

The Statistical Package for Social Sciences version 28 (IBM SPSS, Armonk, New York) and R (R Core Team, 2017) were used for data analysis. All data were tested for normality using Shapiro–Wilk. Demographic differences among the three participant groups were explored using Kruskal–Wallis (age), Mann–Whitney U (lesion size) and chi‐square (sex) tests. Intrarater reliability (IRR) for the FA and diffusivity metrics of the anterior forceps were assessed via an interclass correlation (McGraw & Wong, 1996) using Cronbach's *α*. Nonparametric independent samples (Kruskal–Wallis) tests were performed comparing mean FA, MD, RD, and AD of the anterior and posterior forceps between the AIS, PVI, and control groups along with appropriate pairwise post hoc tests with Bonferroni correction for multiple comparisons. Effect sizes are expressed as eta‐squared (ε^2^). Streamline count was compared among groups using a nonparametric Quade's analysis of covariance (ANCOVA) using estimated total intracranial volume as a covariate. Along‐tract group differences in white matter metrics (FA, MD, AD, RD) were tested using four linear mixed models (LMM). LMMs were performed in R (using the GAMLj module in Jamovi; Jamovi, 2021). Age, group, segment, and the group by segment interaction term were used as fixed factors and patient was used as a random factor in the models. Post hoc tests explored group differences along‐tract segments and multiple comparisons were controlled using the Holm correction.

Spearman's correlations explored associations between age, white matter metrics, and cognitive function. Partial Spearman's correlations (controlling for age) were subsequently used to examine correlations between mean tract FA, MD, RD, and AD of the anterior and posterior forceps of the stroke patients and their parental rating scores from the BRIEF and ADHD‐5 questionnaires. Statistical threshold was set to *p* < .05 and Bonferroni corrected *p*‐value thresholds are given for comparison.

## 3 RESULTS

### 3.1 Population

Initially, 89 participants were recruited, however scans from six children were excluded due to head motion during scanning (AIS *n* = 5; PVI *n* = 1). The final sample thus included 83 participants (AIS *n* = 26; PVI *n* = 26, controls *n* = 31). A subset of stroke participants had cognitive assessments (ADHD: *n* = 20; BRIEF *n* = 19). Age (AIS mean age ± SD = 13.02 ± 4.2 years; PVI age = 11.93 ± 3.5; controls age = 13.25 ± 3.6 years) and sex distribution between groups was comparable (age: *H*_(2) _= 1.65, *p *= .44; sex: χ^2^_(2)_ = 0.79, *p *= .67) and the AIS group had larger lesions than the PVI group (*H*_(1) _= 5.0, *p* < .001). Average time between scanning and cognitive assessment was 2.8 ± 1.47 years (range: 0.48–7.1 years). Additional demographic information and cognitive function scores are presented in Table 1.

**TABLE 1:** Demographic information and parent ratings of cognitive function

	Participant group		
Demographic	AIS	PVI	Controls
Sample (*N*)	26	26	31
Male	16 [61.5%]	16 [61.5%]	16 [51.6%]
Female	10 [38.5%]	10 [38.5%]	15 [48.4%]
Age: mean (SD) [range] years	13.02 (4.15) [6.6–19.5]	11.93 (3.49) [6.6–19.7]	13.25 (3.60) [6.5–19.0]
Stroke side (*N*)			
Left	17 [65.4%]	15 [57.7%]	–
Right	9 [34.6%]	11 [42.3%]	–
Stroke volume: mean (SD) [range] (cc)	44.8 (41.0) [2.9–186.0]	7.9 (13.2) [0.1–50.4]	–
Vascular territory affected (*N*)			
MCA—distal M1	16		
MCA—proximal M1	9		
PCA	1		

**Cognitive function**	**All stroke**	
ADHD‐5 rating scale: %ile (SD) [range]		
Sample size (*N*)	20 (AIS *N* = 13, PVI *N* = 7)	–
Hyperactivity	57.6 (31.6) [5–96]	–
Inattention	67.6 (28.6) [13–99]	–
Total	65.7 (28.3) [18–99]	–
Behavior rating inventory executive function (BRIEF): T scores (SD) [range]		
Sample size (*N*)	19 (AIS *N* = 13, PVI *N* = 6)	–
Inhibit	50.3 (8.2) [37–66]	–
Shift	57.7 (15.0) [36–84]	–
Emotional control	58.4 (12.1) [38–80]	–
Behavior regulation Index	56.1 (11.9) [35–75]	–
Initiate	57.7 (15.5) [36–86]	–
Working memory	60.1 (15.0) [35–87]	–
Plan/organize	59.6 (14.0) [35–89]	–
Organization of materials	53.1 (10.8) [34–71]	–
Monitor	56.0 (12.0) [34–73]	–
Metacognition	59.2 (14.1) [37–88]	–
Global executive composite	58.4 (13.0) [35–77]	–

*Note*: Both the ADHD‐5 rating scale and the BRIEF are negatively scored such that higher scores correspond to more symptoms of ADHD or executive dysfunction.

### 3.2 Intrarater reliability

Intrarater reliability was excellent between the two tractography sessions for the four microstructure variables (FA *α* = .99, MD *α* = .90, RD *α* = .95, AD *α* = .92).

### 3.3 Age correlations

Participant age was moderately correlated with some white matter metrics of both the anterior (MD *r_s_*_ _= −.22, *p *= .045; RD *r_s_*_ _= −.24, *p *= .03) and posterior forceps (FA *r_s_* = .34, *p *= .03) for the entire sample (all groups). When age and WM variables were investigated by participant group separately, controls showed associations for the anterior (FA *r_s_*_ _= .41, *p *= .02; MD *r_s_*_ _= −.38, *p *= .03; RD *r_s_* = −.47, *p *= 0.007) but not posterior forceps. The PVI group showed associations between age and white matter metrics for posterior (FA *r_s_*_ _= .58, *p *= .002; AD *r_s_*_ _= .41, *p *= .04) but not anterior forceps and the AIS group showed no associations with age. For the stroke participants, age was also highly and systematically correlated with higher executive dysfunction as assessed by the BRIEF (Shift *r_s_*_ _= .54, *p *= .02; Behavior Regulation Index *r_s_*_ _= .51, *p *= .03; Initiate *r_s_*_ _= .49, *p *= .03; Plan/Organize *r_s_*_ _= .62, *p *= .005; Organization of Materials *r_s_*_ _= .56, *p *= .01; Monitor *r_s_*_ _= .66, *p *= .002; Metacognition *r_s_*_ _= .65, *p *= .003; Global Executive Composite *r_s_*_ _= .64, *p *= .003) such that older participants showed poorer function. ADHD ratings were not significantly correlated with age.

### 3.4 Differences in white matter metrics among participant groups

**FIGURE 2:**
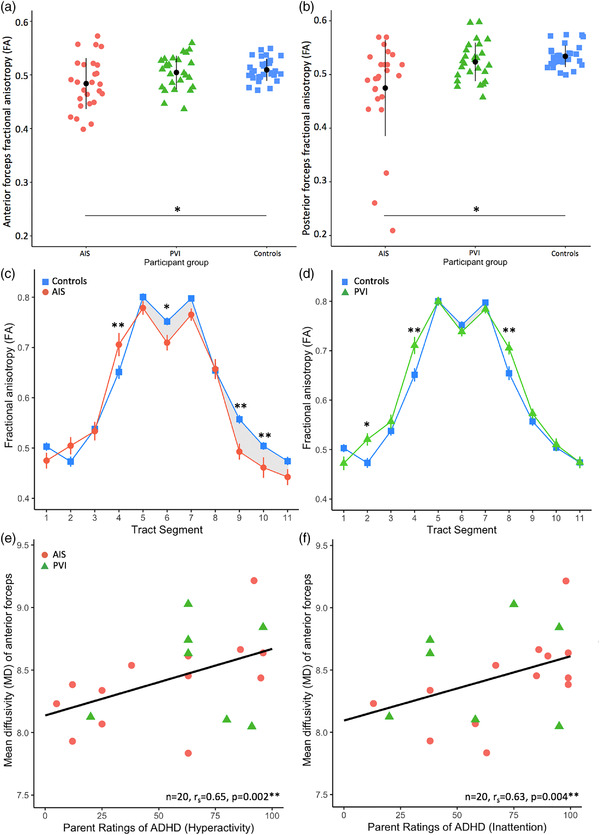
Mean FA for both (a) anterior and (b) posterior forceps showed lower fractional anisotropy (FA) for the AIS group compared to the control group. FA values along the anterior forceps tract varied between (c) AIS vs. controls and (d) PVI vs controls. Shaded areas denote where FA values for the patient group tract segments are lower compared to the control group. Error bars are standard error of the mean. Mean diffusivity of the anterior forceps was associated with parent ratings of (e) hyperactivity and (f) inattention. AIS, arterial ischemic stroke; PVI, periventricular venous infarction. Mean diffusivity is expressed in scientific notation (×10^−4^ mm^2^/s). *p < .05, **p < .01

Anterior and posterior forceps were successfully reconstructed in all participants (Figure 1d–f). As illustrated in Figure 2, mean FA in the anterior forceps was different among groups (*H*_(2) _= 6.3, *p *= .043, ε^2 ^= .08, Table 2) such that FA was lower for AIS compared to controls (*p *= .048). Anterior forceps FA for children with PVI was not different compared to AIS or controls (*p *= .199 and *p *= 1.000, respectively). Streamline count was not different among groups (*F*_(2,80) _= 1.4, *p *= .26). There were no group differences for mean MD, AD, or RD of the anterior forceps (MD: *H*_(2)_ = 1.1, *p *= .58, ε^2^ = .01; AD: *H*_(2)_ = 5.1, *p *= .08, ε^2 ^= .06; RD: *H*_(2) _= 0.3, *p *= .85, ε^2 ^= .004).

**TABLE 2:** Mean white matter metrics by participant group

Structure	Participant group		
White matter metric^a^	AIS	PVI	Controls
Anterior forceps: mean (SD) [range]			
FA	0.48 (0.05) [0.40–0.57]*	0.51 (0.03) [0.44–0.56]	0.51 (0.02) [0.47–0.55]
MD	8.55 (0.6) [7.42–10.2]	8.49 (0.4) [7.60–9.10]	8.57 (0.4) [7.71–9.31]
AD	13.69 (0.7) [12.26–14.95]	13.89 (0.7) [12.8–15.10]	14.06 (0.6) [12.89–15.11]
RD	5.96 (0.7) [4.56–7.75]	5.79 (0.4) [4.80–6.52]	5.82 (0.3) [5.12–6.41]
Posterior forceps: mean (SD) [range]			
FA	0.48 (0.09) [0.21–0.64]**	0.52 (0.04) [0.46–0.60]	0.53 (0.02) [0.50–0.57]
MD	10.1 (2.3) [7.51–19.86]**	8.87 (0.7) [7.47–10.03]	8.62 (0.5) [7.69–9.55]
AD	15.9 (2.9) [12.43–26.34]	14.71 (1.1) [12.54–17.03]	14.32 (0.98) [12.69–15.95]
RD	7.25 (2.2) [4.39–16.62]**	5.97 (0.6) [4.93–7.41]	5.72 (0.4) [5.12–6.57]

AIS, arterial ischemic stroke; PVI, periventricular venous infarction; FA, fractional anisotropy; MD, mean diffusivity; AD, axial diffusivity; RD, radial diffusivity (mm^2^/s).

^a^ MD, AD, and RD are reported in scientific notation (× 10^−4^).

* *p* < .05 compared to controls.

** *p* < .01 compared to controls.

For the posterior forceps, mean FA varied among groups (*H*_(2) _= 11.2, *p *= .004, ε^2 ^= .14). FA for the AIS group was significantly lower compared to controls (*p *= .002) and the FA of the PVI group fell in between AIS and controls, but was not different than either (*p *= .218 and *p *= .422, respectively, Figure 2b, Table 2). Streamline count for posterior forceps showed differences among groups (*F*_(2,80) _= 8.3, *p = *.001), specifically between AIS versus controls (*t*_(80) _= 4.1, *p *< .001), and between AIS versus PVI (*t*_(80) _= 2.4, *p *= .02) such that AIS had fewer streamlines. AIS had higher MD in the posterior region (*H*_(2) _= 17.8, *p *< .001, ε^2 ^= .22) than both the PVI and controls (*p *< .001 and *p *= .016, respectively). MD in the posterior forceps of controls compared to PVI did not differ (*p *= .543). RD (*H*_(2) _= 23.7, *p *< .001, ε^2 ^= .29) was higher in AIS compared to both PVI and controls (*p *= .008 and *p *< .001, respectively) and PVI and controls did not differ (*p *= .229).

### 3.5 Along‐tract white matter metrics

FA varied along the anterior forceps for all groups with the highest values observed at the three midline samples and lowest in more lateral areas (Figure 2c and d). LMM showed significant main effects of group (*F*_(2,83) _= 4.42, *p *= .015), segment (*F*_(10,830) _= 367.8, *p *< .001) as well as a group by segment interaction (*F*_(20,830) _= 3.91, *p *< .001). Age was not a significant main effect (*F*_(1,83) _= .31, *p *= .582) for the FA model.

LMM results for MD showed significant main effects of age (*F*_(1,83) _= 4.93, *p *= .029), group (*F*_(2,83) _= 3.51, *p *= .034), segment (*F*_(10,830) _= 5.18, *p *< .001) as well as a group by segment interaction (*F*_(20,830) _= 2.21, *p *= .002). AD LMM results showed significant main effects of age (*F*_(1,83) _= 7.75, *p *= .007), segment (*F*_(10,830) _= 365.7, *p *< .001) as well as a group by segment interaction (*F*_(20,830) _= 5.18, *p *< .001). RD model results showed significant main effects of group (*F*_(2,83) _= 4.05, *p *= .021), segment (*F*_(10,830) _= 193.4, *p *< .001) as well as a group by segment interaction (*F*_(20,830) _= 3.10, *p *= < .001).

Post hoc tests (Table 3, Figure 2) performed for each along‐tract segment demonstrated group differences in white matter metrics between AIS and controls in the lesioned (segments 9 and 10) and nonlesioned hemispheres (segments 3 and 4) as well as at the midline (segment 6). The PVI group showed largely similar white matter metrics to controls along the anterior forceps except for individual segments in the lesioned (segment 8) and nonlesioned (segment 4).

**TABLE 3:** Holm‐corrected significance *p* values for a long‐tract contrasts between participant groups for four white matter metrics

	Tract segment										
Contrast	Nonlesioned/dominant				Midline				Lesioned/nondominant		
Metric	1	2	3	4	5	6	7	8	9	10	11
AIS vs. controls											
FA	0.117	0.072	0.938	0.001**	0.274	0.015*	0.168	0.295	<0.001**	0.006**	0.214
MD	0.431	0.947	0.002**	0.056	0.088	<0.001**	0.144	0.806	0.021*	0.183	0.463
AD	0.878	0.077	<0.001**	<0.001**	0.240	0.043*	0.395	0.149	0.169	0.205	0.145
RD	0.359	0.306	0.142	0.056	0.160	0.002**	0.026*	0.335	<0.001**	0.018*	0.788
PVI vs. controls											
FA	0.123	0.024*	0.416	0.002**	0.964	0.434	0.573	0.005**	0.442	0.840	0.689
MD	0.510	0.408	0.274	0.303	0.645	0.201	0.875	0.497	0.108	0.188	0.758
AD	0.211	0.242	0.035*	<0.001**	0.476	0.344	0.100	0.050*	0.250	0.218	0.830
RD	0.895	0.082	0.999	0.026*	0.853	0.367	0.407	0.028*	0.197	0.407	0.555
AIS vs. controls											
FA	0.983	0.651	0.480	0.900	0.316	0.112	0.436	<0.001**	<0.001**	0.004**	0.116
MD	0.166	0.391	0.059	0.399	0.233	0.021*	0.122	0.375	<0.001**	0.011*	0.684
AD	0.292	0.567	0.132	0.144	0.659	0.300	0.446	0.001**	0.830	0.975	0.109
RD	0.452	0.489	0.160	0.753	0.244	0.035*	0.181	0.002**	<0.001**	0.002**	0.410

AIS, arterial ischemic stroke; PVI, periventricular venous infarction.

*Note*: Shown are Holm‐corrected *p* values. Shaded cells indicate where FA values were significantly greater for AIS or PVI groups compared to controls.

* *p* < .05.

** *p* < .01.

### 3.6 White matter metrics and cognitive function

A subset of mean anterior forceps white matter metrics were associated with measures of cognitive function (Table 4, Figure 2e and f). Higher ADHD parent ratings were consistently associated with higher diffusivity values such as MD (Hyperactivity *r_s_*_ _= .65, *p *= .002; Inattention *r_s_*_ _= .63, *p *= .004; Total ADHD rating *r_s_*_ _= .61, *p *= .005) and AD (Hyperactivity *r_s_*_ _= .46, *p *= .05; Inattention *r_s_*_ _= .68, *p *= .001; Total ADHD rating *r_s_*_ _= .55, *p *= .014). A subset of these associations remained when the time interval between cognitive assessment and scan was used as an additional covariate (Hyperactivity and AD *r_s_*_ _= .48, *p *= .04; Total ADHD and MD *r_s_*_ _= .48, *p *= .045; Total ADHD and AD *r_s_*_ _= .56, *p *= .02). Correlations between hyperactivity and MD (*r_s_*_ _= .65, *p = *.002) and inattention and AD (*r_s_*_ _= .68, *p = *.001) remained significant after Bonferroni correction. FA of anterior forceps was not associated with ADHD ratings.

**TABLE 4:** Associations between anterior forceps white matter microstructure metrics and clinical executive functioning skills

Questionnaire	White matter metric			
Subtest	FA	MD	AD	RD
ADHD rating scale (*N* = 20)				
Hyperactivity	*r_s_*_ _= .07, *p *= .79	*r_s_*_ _= .65, *p *= .002***	*r_s_*_ _= .46, *p *= .05 *	*r_s_*_ _= .53, *p *= .02 *
Inattention	*r_s_*_ _= −.33, *p *= .17	*r_s_*_ _= .63, *p *= .004 **	*r_s_*_ _= .68, *p *= .001 ***	*r_s_*_ _= .30, *p *= .21
Total	*r_s_*_ _= −.16, *p *= .52	*r_s_*_ _= .61, *p *= .005 **	*r_s_*_ _= .55, *p *= .014 *	*r_s_*_ _= .41, *p *= .07
Behavior rating inventory executive function (BRIEF) (*N* = 19)				
Inhibit	*r_s_*_ _= .45, *p *= .06	*r_s_*_ _= .34, *p *= .17	*r_s_*_ _= .58, *p *= .012 *	*r_s_*_ _= .05, *p *= .85
Shift	*r_s_*_ _= .38, *p *= .12	*r_s_*_ _= .11, *p *= .67	*r_s_*_ _= .34, *p *= .17	*r_s_*_ _= −.23, *p *= .35
Emotional control	*r_s_*_ _= .60, *p *= .009 **	*r_s_*_ _= .19, *p *= .45	*r_s_*_ _= .46, *p *= .06	*r_s_*_ _= −.28, *p *= .26
Behavior regulation Index	*r_s_*_ _= .60, *p *= .008 **	*r_s_*_ _= .23, *p *= .35	*r_s_*_ _= .53, *p *= .02 *	*r_s_*_ _= −.21, *p *= .41
Initiate	*r_s_*_ _= .12, *p *= .64	*r_s_*_ _= .32, *p *= .20	*r_s_*_ _= .42, *p *= .08	*r_s_*_ _= .12, *p *= .62
Working memory	*r_s_*_ _= −.02, *p *= .93	*r_s_*_ _= .51, *p *= .03 *	*r_s_*_ _= .50, *p *= .03 *	*r_s_*_ _= .32, *p *= .20
Plan/organize	*r_s_*_ _= .11, *p *= .67	*r_s_*_ _= .29, *p *= .24	*r_s_*_ _= .34, *p *= .16	*r_s_*_ _= .15, *p *= .56
Organization of materials	*r_s_*_ _= .25, *p *= .33	*r_s_*_ _= .15, *p *= .56	*r_s_*_ _= .32, *p *= .20	*r_s_*_ _= −.01, *p *= .98
Monitor	*r_s_*_ _= .29, *p *= .24	*r_s_*_ _= .21, *p *= .41	*r_s_*_ _= .34, *p *= .16	*r_s_*_ _= −.06, *p *= .83
Metacognition	*r_s_*_ _= .17, *p *= .51	*r_s_*_ _= .31, *p *= .20	*r_s_*_ _= .42, *p *= .08	*r_s_*_ _= .12, *p *= .64
Global executive Composite	*r_s_*_ _= .31, *p *= .22	*r_s_*_ _= .35, *p *= .16	*r_s_*_ _= .54, *p *= .02 *	*r_s_*_ _= .01, *p *= .98

*Note*: Both the ADHD rating scale and the BRIEF are negatively scored such that higher numbers correspond to more symptoms of ADHD and executive dysfunction (i.e., poorer performance). *r_s_*, partial Spearman's correlation controlling for age.

* *p* < .05.

** *p* < .01.

*** *p* < .0036 (Bonferroni correction).

For executive functioning, a subset of BRIEF subscales were associated with mean FA of the anterior forceps (Emotional Control *r_s_*_ _= .60, *p *= .009; Behavior Regulation Index *r_s_*_ _= .60, *p *= .008). Parent ratings of working memory function was also strongly associated with measures of diffusivity (MD [*r_s_*_ _= .51, *p *= .03]; AD [*r_s_*_ _= .50, *p *= .03]) such that poorer white matter metrics were associated with poorer executive function. All of these associations remained when the time interval between cognitive assessment and scan was used as an additional covariate for FA (Emotional Control *r_s_*_ _= .60, *p *= .01; Behavior Regulation Index *r_s_*_ _= .62, *p *= .008) and for MD and AD (Working Memory vs MD *r_s_*_ _= .49, *p *= .04; Working Memory vs. AD *r_s_*_ _= .49, *p *= .046). Additional associations were identified between BRIEF subscales and AD (Inhibit *r_s_*_ _= .57, *p *= .02; Behavioral Regulation Index *r_s_*_ _= .52, *p *= .03; Global Executive Composite *r_s_*_ _= .53, *p *= .03). No associations between white matter metrics and BRIEF executive function ratings were observed after Bonferroni correction.

None of the posterior forceps mean white matter metrics showed significant correlations with the ADHD or BRIEF measures.

## 4 DISCUSSION

Using dMRI tractography, we have shown group differences in underlying microstructure of the forceps in children with perinatal stroke compared to controls. Specifically, children with AIS showed disrupted white matter metrics in both anterior and posterior forceps compared to those with periventricular venous infarction and controls. Along‐tract analyses showed spatially specific differences in metrics suggestive of remote diaschisis and possible compensatory neuroplasticity in the nonlesioned hemisphere. Frontal (but not posterior) white matter metrics were associated with parental ratings of ADHD symptoms, such that higher white matter diffusivity values were associated with poorer function. These findings suggest that underlying microstructure of frontal white matter quantified via tractography can provide a relevant biomarker of attention and hyperactivity behaviors in children with perinatal stroke.

### 4.1 Anterior forceps

Using dMRI tractography to isolate the anterior forceps in the frontal lobe, we demonstrated that underlying microstructure (i.e., fractional anisotropy) of frontal white matter in AIS participants appeared to be altered. This finding is consistent with previous literature investigating frontal white matter in unilateral cerebral palsy reporting lower FA values in anterior cingulate cortex and superior frontal gyrus (Scheck et al., 2015). This is also consistent with the wider ADHD literature that demonstrates lower FA values and higher diffusivity values in frontal white matter of children with ADHD (Konrad & Eickhoff, 2010; Liston et al., 2011; Tremblay et al., 2020; van Ewijk et al., 2012). Given that these reports use mean metrics extracted from large white matter structures, it is compelling that group differences can still be detected despite using methods with relatively low spatial resolution.

### 4.2 Along‐tract analyses and secondary degeneration

Using along‐tract analyses, we found spatially specific differences in microstructure metrics in the frontal white matter of children with AIS. Along‐tract analyses provided more spatially specific quantification of the spatial extent of possible diaschisis and Wallerian degeneration in areas displaced from the primary lesion allowing for sensitive group comparisons evidenced by significant group by segment interactions in linear mixed models. Our findings suggest that in addition to primary stroke damage to lateral frontal white matter, secondary degeneration of more medial white matter may also underlie group differences in frontal tracts. Such secondary damage of the motor network via diaschisis (Craig et al., 2019; Craig, Olsen et al., 2019; Srivastava et al., 2019) and/or Wallerian degeneration (De Vries et al., 2005; Kirton et al., 2007) has been previously documented in this population. In the current study, some children showed white matter metric values similar to controls while others showed large departures, supporting the idea of heterogeneous and patient‐specific secondary degeneration of connected structures in cognitive networks.

### 4.3 Compensatory neuroplasticity

Our findings also highlight areas in the nonlesioned hemisphere that showed *higher* group mean FA values in both stroke groups compared to controls. This may relate to compensatory developmental neuroplasticity in the nonlesioned hemisphere, though larger studies are needed to more specifically explore associations with cognitive function. Initial studies in this population have demonstrated differences in thalamic (Craig, Carlson et al., 2019) and cerebellar volumes (Craig, Olsen et al., 2019) as well as more complex differences in graph theory metrics quantifying overall structural connectivity of the nonlesioned hemisphere (Craig et al., 2020). Such metrics have been strongly associated with motor function, again suggesting functionally relevant compensatory processes in the nonlesioned hemisphere. We have also documented spatially specific differences in cortical thickness/volumes, gyrification, and sulcal depth (Shinde et al., 2021) as well as myelination (Yu et al., 2018) in the nonlesioned hemisphere after perinatal stroke. Given the heterogeneity of direct stroke‐induced damage and the possibility of varying degrees of remote diaschisis and/or neuroplastic compensation in the nonlesioned hemisphere, it is perhaps not surprising that there is large variability in cognitive functioning within this group.

Group differences in frontal white matter were not as apparent in children with PVI, possibly because PVI‐induced damage is largely restricted to subcortical periventricular white matter rather than cortex. Because PVI occurs earlier in brain development, there may also be more opportunity for neuroplastic compensation. Indeed, for three regions of the anterior forceps, the PVI group appeared to have *higher* FA of frontal white matter compared to controls. Children with PVI are more likely to show motor disabilities (especially of the lower limb) rather than cognitive disabilities given the periventricular location of primary white matter damage and cortical sparing (Dunbar & Kirton, 2019; Kirton et al., 2008). Consistent with this, our sample contained fewer PVI participants (*n* = 6) than AIS (*n* = 14) referred for clinical neuropsychological assessment reflecting relatively intact cognition and fewer concerns worthy of referral.

### 4.4 ADHD

Associations between structure and function add additional clinical interest. It is now well established that children with perinatal stroke show a higher prevalence of ADHD compared to children without neurologic injury (Bosenbark et al., 2018; Fuentes et al., 2016; Kirton & deVeber, 2013; Murias et al., 2014; Thomas et al., 2015). We have demonstrated systematic associations between frontal white matter microstructure disruptions (higher diffusivity values) and parent ratings of hyperactivity, inattention, and total ADHD scores consistent with previous studies (Konrad & Eickhoff, 2010; Lawrence et al., 2013; Liston et al., 2011). Lower diffusion anisotropy, leading to higher MD, AD and RD values, is thought to reflect more unrestricted diffusion via underlying differences in axonal membranes, neurofibrils, or cellular density which may in turn be modulated by degree of myelination (Beaulieu, 2002). The apparently disrupted white matter microstructure in frontal anterior forceps as measured by three diffusivity variables appears to be associated with higher parental ADHD ratings. These findings are consistent with previous literature reporting higher diffusivity values in children with ADHD (Liston et al., 2011; van Ewijk et al., 2012) as well as correlations with parental reports of inattention (Lawrence et al., 2013).

Although MD values showed significant correlations with ADHD ratings, FA did not. It could be that since FA is a complex ratio between diffusion in multiple orientations, it provides a less specific quantification of underlying microstructure compared to other, more directional, microstructure metrics. Further, FA is more affected by underlying white matter structural organization (such as crossing fibers) compared to diffusivity values, which may also contribute to this disparity (van Ewijk et al., 2012). Previous studies investigating ADHD have reported this same pattern where diffusivity metrics show functional correlations and FA does not (Lawrence et al., 2013).

### 4.5 Executive function

Strong correlations were found between age and executive functioning in our perinatal stroke sample despite the use of scaled scores expressed in relation to age‐matched normative values. Deficits in cognitive functioning may not become apparent until later in development as parental expectations change as children age (Bosenbark et al., 2018). However, this age correlation could alternatively reflect an altered frontal white matter developmental trajectory for children with perinatal stroke. If the typical trajectory of cognitive development has been arrested, delayed, or prolonged due to early stroke‐induced damage (Westmacott et al., 2009), more deficits in executive function may become apparent as children age. These may manifest as strong correlations between age and parental ratings of dysfunction. Specifically, deficits in children with AIS compared to controls have previously been noted in measures of attention, verbal retrieval, inhibitory control, flexibility/shifting, planning/organization, and processing speed (Bosenbark et al., 2018). As a group, our perinatal stroke sample showed broadly normal executive functioning as indicated by group average measures; however, a very wide range was observed on all subscales measured. Clearly, cognitive consequences of direct and secondary damage due to stroke are heterogeneous, varying widely among patients.

Parental ratings of executive functioning did not appear to be as highly associated with anterior forceps white matter metrics as ADHD ratings. Although some correlations were fairly strong (*r_s_* > .5), many associations did not reach statistical significance. MD and AD showed positive correlations with working memory whereas AD had positive associations with measures of inhibition, behavioral regulation index, and global executive composite. Taken together, this reveals a pattern of poorer ability to regulate behavior corresponding to disruptions in underlying microstructure of frontal white matter. This is consistent with our current findings of higher ratings of inattention and hyperactivity on the ADHD rating scale and previously reported difficulties with inattention after perinatal AIS (Bosenbark et al., 2018).

There was an absence of significant correlations between white matter metrics and measures of metacognition such as planning, organizing, and monitoring, although prior studies have suggested associations in children with AIS (Bosenbark et al., 2018). It could be that these deficits do occur in this population, but are not associated with white matter metrics of frontal white matter alone. These patterns may be better elucidated by studies examining more frontal white matter tracts beyond anterior forceps, as well as other neuroimaging markers such as white matter volumes, cortical thickness of frontal cortical areas such as dorsolateral prefrontal cortex (DLPFC), or functional connectivity measures of wider attentional networks.

One unexpected finding of the current study was the absence of widespread negative correlations between FA values and executive functioning. Although other studies have reported this same finding (Scheck et al., 2015), we expected to find that higher FA in frontal white matter would correspond to better function. Further, the two associations we did identify with emotional control and behavioral regulation index were in the strongly positive direction such that higher FA was associated with poorer executive function. As mentioned above, FA is a composite ratio of multiple diffusion orientations and is influenced by many factors such as degree of axonal packing, presence of microtubules, myelination, and crossing fibers (Beaulieu, 2002). The reasons underlying the absence of negative correlations remain unclear; however, they may be related to the sensitivity of FA to multiple underlying tissue properties, its nonspecificity, and the apparent heterogeneity of FA–behavior relationships (Lazari et al., 2020).

### 4.6 Posterior forceps

As hypothesized, the posterior forceps (our comparison tract) appeared to be functionally independent, showing no correlations with executive dysfunction and/or ADHD symptoms. This finding does suggest that differences in frontal white matter after perinatal stroke are functionally specific, at least for the measures presented here, and do not appear to be associated with differences in posterior white matter. Interestingly, posterior white matter did show group differences in volume and underlying microstructure for the AIS group compared to controls. This could be due to direct stroke‐induced damage since infarctions of the middle cerebral artery can directly damage posterior visual areas in some cases. In children without direct lesion damage, it also supports the concept of secondary degeneration of areas displaced from primary stroke damage as may be the case with the anterior forceps. These differences also suggest possible deficits in functions mediated by the dorsal visual stream and projections to parietal sensory integration areas after perinatal stroke (Knyazeva, 2013).

### 4.7 Limitations

Certain limitations of the current study must be acknowledged. ROI placement for tract reconstruction was manually performed by one tractographer and thus may have been somewhat subjective compared to automated techniques. This is a ubiquitous challenge for studies using manual tractography but is necessary in this population since the brain anatomy following stroke is typically displaced rendering automated and atlas‐based tractography methods ineffective. This limitation was somewhat mitigated by our excellent intrarater reliability of microstructure values. We used tensor‐based metrics for white matter microstructure quantification, which are more conservative in streamline selection compared to more complex techniques such as constrained spherical deconvolution (CSD) (Tournier et al., 2004). In addition, using a higher b‐value, multishell acquisitions, and better eddy‐current distortion corrections may have been more sensitive in detecting differences in WM metrics. Further, more directly modeling free water components of the diffusion signal (Pasternak et al., 2009) would have likely provided additional insight into underlying anatomical architecture. Along‐tract segments were in approximately the same position for each participant given that sampling occurred in patient space. Head motion is a challenge for pediatric neuroimaging, especially for participants who may have attentional and cognitive disorders. Those unable to successfully complete an MRI had their scans discarded, ultimately leaving an underrepresentation of more severe cases.

Participants with AIS were overrepresented compared to PVI in the subset of participants with cognitive testing as they were part of a cohort referred for clinical neuropsychological assessment. Comparative measures of ADHD and executive function were not acquired for the control group though population‐based normative scales were available for calculating percentiles and T scores. ADHD and executive functioning for the stroke groups were based on parental report, which is informative but may be prone to subjectivity of such parent report. Prospective, standardized cognitive testing of participants themselves could have been utilized to more directly measure cognitive function and may have provided different associations with white matter metrics. The timing between cognitive assessments and MRI was greater than one year. As neuroimaging metrics can change with experience and age, caution should be taken in interpreting this association when measures are taken at different times.

## 5 CONCLUSION

Based on diffusion tensor imaging‐based tractography, spatially specific differences in underlying microstructure of the anterior forceps occur in children with perinatal stroke and are associated with stroke type and behavioral outcomes of executive function and attention. Furthering our understanding of associations between cognitive outcomes and the associated underlying brain differences that accompany them may facilitate the advancement of biomarker identification.

## FUNDING

This study received funding from the Canadian Institutes of Health Research (CIHR) and the Heart and Stroke Foundation of Canada. NL received funding from Alberta Innovates. BTC received funding from a Vanier Graduate Scholarship. BLB receives salary funding from the Canadian Institutes of Health Research (CIHR) Embedded Clinical Researcher Award.

## DISCLOSURES

Dr. Brooks receives royalties for the sales of the *Pediatric Forensic Neuropsychology* textbook (2012, Oxford University Press) and three pediatric neuropsychological tests (not used in the current study) (Child and Adolescent Memory Profile [ChAMP, Sherman and Brooks, 2015, PAR Inc.], Memory Validity Profile [MVP, Sherman and Brooks, 2015, PAR Inc.], and Multidimensional Everyday Memory Ratings for Youth [MEMRY, Sherman and Brooks, 2017, PAR Inc.]).

### PEER REVIEW

The peer review history for this article is available at https://publons.com/publon/10.1002/brb3.2433

## Data Availability

The data that support the findings of this study are available from the corresponding author upon reasonable request.
